# Effect of Smear Layer on the Push-Out Bond Strength of Two Different Compositions of White Mineral Trioxide Aggregate

**Published:** 2013-10-07

**Authors:** Mehrdad Lotfi, Saeed Rahimi, Negin Ghasemi, Sepideh Vosoughhosseini, Mahmood Bahari, Mohammad Ali Saghiri, Atabak Shahidi

**Affiliations:** a Department of Endodontics, Dental School, Tabriz University of Medical Sciences, Tabriz, Iran;; b Department of Oral and Maxillofacial Pathology, Dental School, Tabriz University of Medical Sciences, Tabriz, Iran;; c Department of Operative Dentistry, Dental School, Tabriz University of Medical Sciences, Tabriz, Iran;; d Research Associated, Department of Ophthalmology and Visual Sciences, University of Wisconsin School of Medicine and Public health, Madison, WI, USA;; e Research Scientist, Orumieh, Iran

**Keywords:** Disodium Hydrogen Phosphate, Mineral Trioxide Aggregate, Push-Out Bond Strength, Smear Layer, Root Canal Preparation

## Abstract

**Introduction:**

The aim of this in vitro study was to evaluate the effect of smear layer on the push-out bond strength of white mineral trioxide aggregate (WMTA) with and without disodium hydrogen phosphate (Na_2_HPO_4_).

**Materials and Methods:**

Dentin discs with standard cavities were obtained from extracted human single-rooted teeth and divided to 4 groups (n=15) according to the irrigation regimen and the canal filling material. In groups 1 and 3, canals were irrigated with normal saline; in groups 2 and 4, irrigation method included sodium hypochlorite (NaOCl) and then ethylenediaminetetra-acetic acid (EDTA). The canals were filled with WMTA in first and second groups and with WMTA+Na_2_HPO_4_; in groups 3 and 4. The samples were wrapped in wet gauze and incubated in 37°C for 3 days. The push-out bond strength was then measured by means of the *Universal Testing Machine* and the failure modes were examined under stereomicroscope at 40× magnification. Tow-way ANOVA was used to evaluate the effect of material type and smear layer removal. Post hoc Tukey test was used for the two-by-two comparison of the groups.

**Results:**

The greatest and lowest mean±standard deviation for push-out bond strength were observed in groups 4 (4.54±1.14 MPa) and 1 (1.44±0.96 MPa), respectively. The effect of removing the smear layer on the push-out bond strength of WMTA+Na_2_HPO_4_ was significant (*P*=0.01), but not for WMTA (*P*=0.52). Interestingly, there was significant difference between groups 1, 3 and 2, 4 (*P*<0.05). The failure mode for all experimental groups was of mixed type.

**Conclusion:**

Under circumstances of this in vitro study, removal of smear layer increases push-out bond strength when Na_2_HPO_4_ is added to WMTA.

## Introduction

Mineral trioxide aggregate (MTA) is a mixture of dicalcium silicate, tricalcium silicate, tricalcium aluminate, tetracalcium aluminoferrite, and bismuth oxide (1). It has been widely used for perforation repair, root canal treatment of immature teeth and as a root-end filling material during apical surgery (2, 3). MTA has met most of the requirements of an ideal root-end filling material. However, subcutaneous implantation of MTA in rats provokes severe initial reactions with coagulation necrosis and dystrophic calcification. Apart from its initial inflammatory cell reactions, its working and setting time are not ideal. (4-9). One approach to reduce the setting time is to use disodium hydrogen phosphate (Na_2_HPO_4_) as an accelerator (10). Huang *et al*. (11) , showed that 15% Na_2_HPO_4_ solution as a liquid phase can reduce the setting time of white MTA (WMTA) to 26 min, and a diametral tensile strength of 4.9 MPa at the initial 6-hour period can be achieved. They suggested that Na_2_HPO_4_ solution might be an effective setting accelerator for WMTA. Ding *et al*. (12), suggested that Na_2_HPO_4_ solution as an MTA accelerator reduces the setting time and maintains the pH value. In addition, Lotfi *et al*. showed that adding Na_2_HPO_4_ to WMTA creates a more biocompatible material than WMTA alone (13).

Considering the clinical applications of MTA, the bond strength of material with dentin is an important factor in achieving the best sealing ability. In other words, in cases of root-end filling, the material need to remain in desired place and tolerate dislodging forces such as functional loads. The push-out bond test aims to assess the bond strength of the materials to dentin (14, 15).

The smear layer which contains organic and inorganic components is formed on dentinal walls during root canal preparation. The presence of such a layer compromises the penetration of root canal irrigants and the obturating materials into dentinal tubules, which increases the risk of bacterial infection and microleakage (16). Thus, the removal of smear layer can improve the adaptation of root canal filling materials (17). Yildirim *et al*. showed that the apical microleakage of MTA is less in the absence of the smear layer (18).

**Table 1.** The mean (SD) deviation of push-out in experimental groups

Groups	Materials	Smear layer	Strength
**1**	WMTA	Removed	1.44 (0.96) MPa
**2**	WMTA	-	2.17 (0.72) MPa
**3**	WMTA+Na2HPO4	Removed	2.98 (1.56) MPa
**4**	WMTA+Na2HPO4	-	4.54 (1.51) MPa

Considering the absence of studies regarding the push-out bond strength of WMTA with and without Na_2_HPO_4_, the purpose of this *in vitro* study was to evaluate the effect of smear layer on the push-out bond strength of these two compositions of WMTA.

## Material and Methods

Sixty extracted single-rooted human maxillary anterior teeth were selected for this study. The teeth were stored in 0.5% chloramine-T. 3 mm thick slices of dentin were prepared from the mid root area using water-cooled diamond disc (SP1600 microtome; Leica, Nußloch, Germany). The lumen of the dentin slices were drilled with #2 to #5 (ISO sizes #70 to 130) Gates-Glidden burs (Dentsply, Maillefer, Ballaigues, Switzerland) to obtain 1.3 mm diameter cavities.

The samples were randomly divided into 4 groups; then they were mounted on slaps by using sticky wax to simulate the close-end model. All specimens were checked to ensure the absence of any penetration pathway for irrigation solution out of the lumen. In groups 1 and 3 irrigation was performed using 2 mL of normal saline and the cavity was dried by size #90 paper cone (Aryadent, Tehran, Iran) without contacting with canal walls. In groups 2 and 4, the standard smear layer removal method was conducted, using 2 mL 5.25% sodium hypochlorite (NaOCl) for 10 min then 2 mL normal saline followed by 17% ethylene diamine tetra acetic acid (EDTA) for 5 min were used. Two milliliters of distilled water was applied as a final flush and the canals were dried with paper cones as previously described.

In groups 1 and 2, WMTA (Tooth-colored Formula) (Dentsply, Tulsa Dental, Tulsa, OK) was mixed with distilled water according to the manufacturer’s instructions and placed into the lumens. In group 3 and 4, WMTA was mixed with 2.5 wt% of Na_2_HPO_4_ (Merck, Darmstad, Germany), then the powder was mixed with distilled water with a 0.3 mL/g. liquid/powder ratio. Then the lumens were filled. The samples were wrapped in gauze, wet with distilled water and incubated for 3 days in 37°C.

After the experimental time period, the samples were submitted to the push-out bond test. The push-out bond strength was measured by using *Universal Testing Machine* (Hounsfild Test Equipment, Model H5k-s, Surrey, England). The WMTA was loaded with a 1 mm diameter cylindrical stainless steel plunger at a speed of 1 mm per min. The maximum load applied to the material, which was displayed in Newton, was recorded just before dislodgment. To express the value in MPa, the recorded amount in Newton was divided by area in mm^2^ according to the following formula: 2πr×h, where the π is the constant 3.14, *r* is the root canal radius, and then *h* would be the thickness of the root slice in millimeters. The slices were then examined under the stereo-microscope at 40× magnification to determine the mode of bond failure. Each sample was placed into 1 of the 3 failure modes: *adhesive failur*e that occurred at the material and dentin interface, *cohesive failure* that happened within the material, and *mixed failure* mode.

Two-way ANOVA was used to evaluate significance of the effect of material type and smear layer removal. Post hoc Tukey tests were used for the two-by-two comparison of the groups. Statistical significance was defined at *P*<0.05. SPSS 18 statistical software was used for the analysis of data.

## Results

The greatest and lowest mean±standard deviations for push-out bond strength were observed for groups 4 (4.54±1.14 MPa) and 1 (1.44±0.96 MPa), respectively (Table 1).The effect of smear layer removal on push-out bond strength of WMTA+Na_2_HPO_4 _was significant (between groups 3 and 4; *P*=0.01). However, it was not significant for WMTA (between groups 1 and 2; *P*=0.52). Interestingly, there was significant difference between groups 1, 3 and 2, 4 (*P*<0.05). The failure mode for all tested groups was of mixed type. 

## Discussion

This study was designed to compare the push-out bond strength of WMTA and WMTA+Na_2_HPO_4_, and also to assess the effect of smear layer removal on the dislodgment resistance of these two compositions. Endodontic materials like MTA should be able to remain in place under dislocating forces such as functional stresses caused by tooth movement during mastication or operative procedures (14, 15). Therefore, the push-out strength of the materials that are used for vital pulp therapy, apical barrier and as root-end filling material is important to achieve successful treatment. There are different methods for measuring the adhesion of a dental material to dentin including tensile, shear, and push-out bond strength tests. The push-out test has been used in this study as a reliable method (14).

Recently some efforts were done to improve the properties of MTA. One of which was addition of Na_2_HPO_4_ to reduce its setting time (11-13). Adding Na_2_HPO_4_ to WMTA can promote HA formation of hydroxyapatite, due to presence of amorphous calcium phosphate phase (11-13).

Sarkar *et al*. (19), suggested that MTA releases calcium hydroxide after mixing with water which interacts with a phosphate-containing fluid to produce calcium-deficient apatite via an amorphous calcium phosphate phase (20). Thus, hydroxyapatite crystals nucleate and grow, filling the microscopic spaces between MTA and dentinal walls. Besides verifying the presence of HA interfacial layer, a study reported the formation of *Tag-Like Structures* (TS) extending from the intermediate layer to the dentinal tubules, similar to those reported at the resin-dentin interface .This interlocking, improves the mechanical retention of the material that is used as an end plug in root canal space (21, 22). Our study showed that the effect of smear layer removal was not significant even in presence of Na_2_HPO_4_ added to WMTA. This result may be explained by the fact that WMTA cannot produce HA in presence of water. Thus, open dentinal tubules were not effectively filled by *HA*.

Increased push-out bond strength in the recent study after adding Na_2_HPO_4_ to WMTA could be explained by formation of *TS* within dentinal tubules. However, push-out bond strength did not increase when smear layer was not removed. In other words, in spite of HA formation, smear layer acts as a barrier and prevents TS formation within dentinal tubules. In addition, Reyes-Carmona *et al*. (21-23), revealed that the formed apatite crystals deposited within collagen fibrils, which promoted the controlled mineral nucleation on dentin and triggered the formation of an interfacial layer with TS at the cement-dentin interface that improved sealing ability.

From a clinical point of view, the biomineralization process which is supported by the interaction of biomaterials with dentin in presence of phosphate groups, and removal of smear layer to open dentinal tubules; both together boost the resistance to displacement of the biomaterials from dentin. This process could be responsible for the superior dislodgement resistance in WMTA+Na_2_HPO_4_.

## Conclusion

Removal of smear layer increases push-out bond strength when Na_2_HPO_4_ is added to WMTA.
